# Involvement of Ras GTPase-activating protein SH3 domain-binding protein 1 in the epithelial-to-mesenchymal transition-induced metastasis of breast cancer cells via the Smad signaling pathway

**DOI:** 10.18632/oncotarget.3636

**Published:** 2015-04-20

**Authors:** Hao Zhang, Yan Ma, Shenghua Zhang, Hong Liu, Hongwei He, Naren Li, Yuyan Gong, Shuangshuang Zhao, Jian-dong Jiang, Rong-guang Shao

**Affiliations:** ^1^ Department of Oncology, Institute of Medicinal Biotechnology, Peking Union Medical College, Chinese Academy of Medical Sciences, Beijing, China; ^2^ Institute of Medicinal Biotechnology, Peking Union Medical College, Chinese Academy of Medical Sciences, Beijing, China; ^3^ State Key Laboratory of Bioactive Substances and Functions of Natural Medicines, Institute of Materia Medica, Peking Union Medical College, Chinese Academy of Medical Sciences, Beijing, China

**Keywords:** G3BP1, EMT, breast cancer, Smad signaling pathway

## Abstract

In situ models of epithelial-to-mesenchymal transition (EMT)-induced carcinoma develop into metastatic carcinoma, which is associated with drug resistance and disease recurrence in human breast cancer. Ras GTPase-activating protein SH3 domain-binding protein 1 (G3BP1), an essential Ras mediator, has been implicated in cancer development, including cell growth, motility, invasion and apoptosis. Here, we demonstrated that the upregulation of G3BP1 activates the EMT in breast cancer cells. Silencing Smads almost completely blocked this G3BP1-induced EMT, suggesting that this process depends on the Smad signaling pathway. We also found that G3BP1 interacted with the Smad complex. Based on these results, we proposed that G3BP1 might act as a novel co-factor of Smads by regulating their phosphorylation status. Moreover, knockdown of G3BP1 suppressed the mesenchymal phenotype of MDA-MB-231 cells *in vitro* and suppressed tumor growth and lung metastasis of 4T1 cells *in vivo*. Our findings identified a novel function of G3BP1 in the progression of breast cancer via activation of the EMT, indicating that G3BP1 might represent a potential therapeutic target for metastatic human breast cancer.

## INTRODUCTION

In recent years, breast cancer has become the most common and fatal cancer among women worldwide [1]. The predominant cause of death and obstacle to successful treatment of this disease is metastasis. Because the epithelial-to-mesenchymal transition (EMT) can convert benign tumors into invasive and metastatic tumors, it plays a critical role in the regulation of tumor progression and metastasis [2–4]. The EMT is a developmental process that enables polarized epithelial cells to undergo multiple biochemical changes to exhibit the phenotypes of mesenchymal cells, including enhanced migratory and invasive capacities and elevated resistance to apoptosis [2].

Many signaling pathways participate in the EMT, including the TGF-β, Notch, Ras, Wnt and Hedgehog signaling pathways [5, 6], and the Smad signaling is necessary for TGF-β-induced EMT. Smads interact with EMT-promoting transcription factors that comprise epithelial repressors, such as Snail, Twist and Zeb, and mesenchymal activators (MeA), including β-catenin, AP-1 and TCF, resulting in the formation of EMT-promoting Smad complexes (EPSCs) to induce the EMT [7]. Moreover, ectopic Smad2 or Smad3, in combination with Smad4, enhances TGF-β-induced EMT, whereas dominant-negative Smad2, Smad3, or Smad4 blocks the TGF-β-induced EMT in NMuMG breast epithelial cells [8, 9].

G3BP1 was initially identified as a ubiquitously expressed cytosolic 68 kDa protein that co-immunoprecipitates with the GAP SH3 domain [10]. Because RasGAP is the primary negative regulator of Ras and because the RasGAP SH3 domain is important for oncogenic Ras signaling pathways [11], G3BP has been implicated in regulation of the Ras signaling pathway. Both G3BP1 and G3BP2 are dramatically overexpressed in human cancers, especially breast cancer, suggesting that they may be related to cancer progression [12, 13]. Recent evidence has suggested that the overexpression of the N-terminal of G3BP enhanced the motility and invasion of PDAC cells [14, 15]. Previously, we demonstrated that G3BP knockdown inhibited HCT116 cell growth via the induction of apoptosis [16]. Furthermore, we reported that G3BP knockdown inhibited the migration and invasion of H1299 cells by suppressing the Src/FAK-associated signaling pathway [17]. This evidence suggested that G3BP1 may participate in the regulation of cell invasion and migration.

Therefore, in this study, we hypothesized that G3BP1 participates in the EMT to induce tumor metastasis in human breast cancer. Because G3BP1 is overexpressed during breast cancer, we aimed to investigate whether G3BP1 was involved in the EMT and to elucidate the mechanism by which G3BP1 regulates tumor metastasis in human breast cancer.

## RESULTS

### Overexpression of G3BP1 induces the EMT in MDCK cells

**Figure 1:**
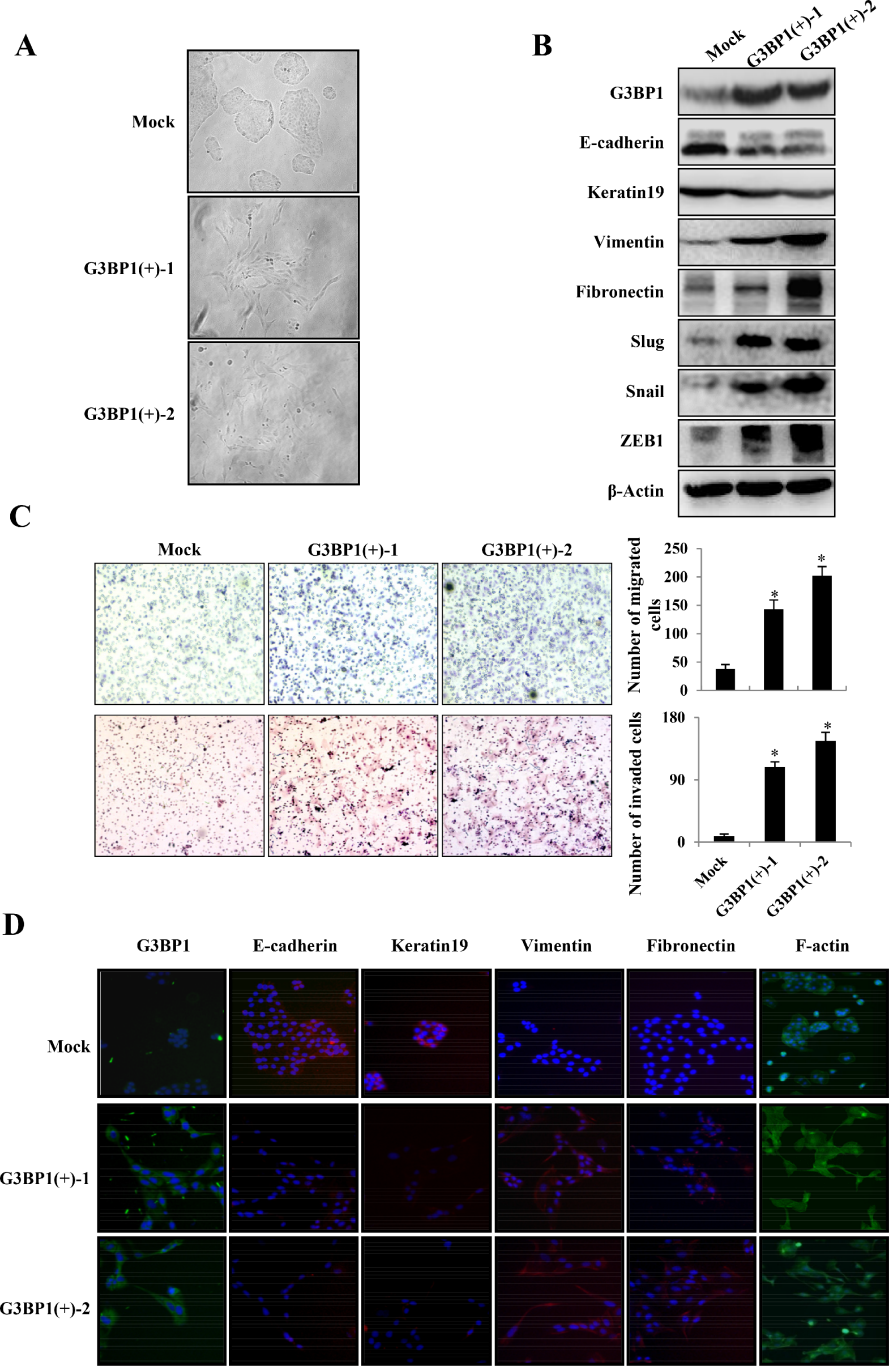
Stable overexpression of G3BP1 induces the EMT and enhances the migration and invasion of MDCK cells **A.** Morphological changes in MDCK cell lines stably expressing G3BP1 (G3BP1(+)-1 or G3BP1(+)-2) or an empty vector (Mock) (magnification, × 100). **B.** Western blot of whole-cell extracts from MDCK cells (Mock, G3BP1(+)-1 or G3BP1(+)-2) for G3BP1, E-cadherin, Keratin, Fibronectin, Vimentin, Snail, Slug and Zeb1. β-actin was used as a loading control. **C.** Transwell migration assay and Matrigel invasion assay using MDCK cells (Mock, G3BP1(+)-1 or G3BP1(+)-2) (magnification, × 100). *P < 0.05 versus Mock. The data represent the means±s.d. of three independent experiments. **D.** The expression of G3BP1, E-cadherin, Keratin19, Fibronectin, Vimentin and F-actin was detected via immunofluorescence staining (magnification, × 100).

Polarized MDCK epithelial cells converted into migratory fibroblasts in response to the addition of conditioned medium from cultured fibroblasts; this method has been used extensively as a model of the EMT *in vitro*[18]. Therefore, we employed this model to investigate whether overexpression of G3BP1 could induce EMT and enhance the migration and invasion of MDCK cells. By transfecting with pCDNA3.1-G3BP1, we generated two stably G3BP1-overexpressing MDCK cell lines that displayed increased expression of G3BP1 compared with MDCK cells transfected with the pCDNA3.1-Mock vector (Figure 1B and 1D). Concomitant with the increase in G3BP1 expression, characteristic morphological changes associated with the EMT were observed in the G3BP1(+) clones. The stably G3BP1-overexpressing cells exhibited a spindle-like rather than cobblestone-like fibroblast morphology (Figure 1A). Moreover, the two stably G3BP1-overexpressing cell lines grew despite the lack of intercellular tight junctions, indicating a mesenchymal-like phenotype, whereas the Mock vector-transfected cells maintained their cobblestone-like phenotype in the presence of strong cell-cell adhesions.

Our results also demonstrated that G3BP1 promoted MDCK cell migration and invasion (Figure 1C). Enhanced migratory capacity and invasiveness characterize cells that have undergone the EMT. To address whether G3BP1 induces the EMT, we then examined the levels of EMT markers via western blot and immunofluorescence staining. As shown in Figure 1B and 1D, expression of the epithelial cell-cell adhesion molecule E-cadherin and the intermediate filament protein Keratin 19 was dramatically decreased, whereas the mesenchymal markers Fibronectin and Vimentin were significantly upregulated in G3BP1-overexpressing clones. Several transcription factors, such as SIP1, Snail1 (Snail), Snail2 (Slug), and ZEB1, inhibit E-cadherin expression, which serves as a key event in the disruption of tight cell-cell contacts, thereby activating the EMT. Consistently, overexpression of G3BP1 increased the levels of Snail, Slug and ZEB1. Taken together, our data indicated that overexpression of G3BP1 induced the EMT in MDCK cells.

### Overexpression of G3BP1 induces the EMT in MCF-7 cells

**Figure 2:**
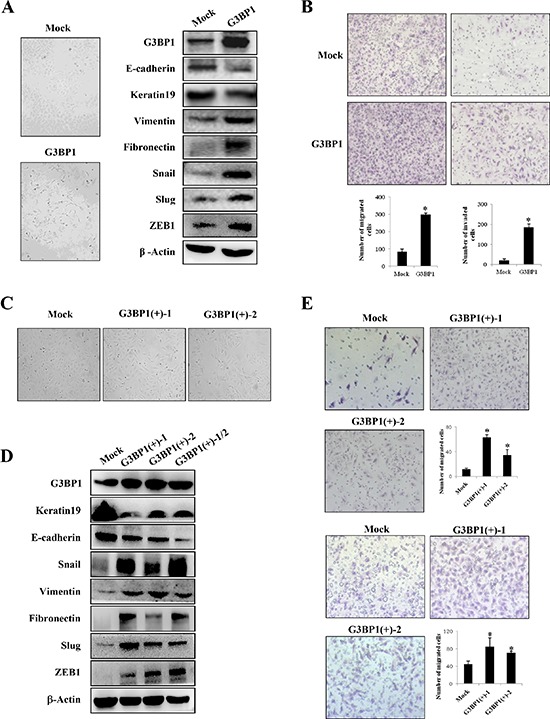
Overexpression of G3BP1 induces the EMT and enhances the migration and invasion of MCF-7 cells **A.** Changes in the morphology (left) and expression of epithelial and mesenchymal markers (right) of MCF-7 cells transiently transfected with pCDNA3.1-G3BP1 (G3BP1) or pCDNA3.1-control (Mock) (magnification, × 100). β-actin was used as a loading control. **B.** Transwell migration assay (left) and Matrigel invasion assay (right) using MCF-7 cells (Mock or G3BP1) (magnification, × 100). *P < 0.05 versus Mock. The data represent the means±s.d. of three independent experiments. **C.** Morphological changes in MCF-7 cell lines stably expressing G3BP1 (G3BP1(+)-1 or G3BP1(+)-2) or an empty vector (Mock) (magnification, × 100). **D.** Western blot of whole-cell extracts from MCF-7 cells (Mock, G3BP1(+)-1,G3BP1(+)-2 or the mixture of the two clones (G3BP1(+)-1/2)) for epithelial and mesenchymal markers. β-actin was used as a loading control. **E.** Transwell migration assay (upper) and Matrigel invasion assay (lower) using MCF-7 cells (Mock, G3BP1(+)-1 or G3BP1(+)-2) (magnification, × 100). *P < 0.05 versus Mock. The data represent the means±s.d. of three independent experiments.

To investigate the role of G3BP1 in breast cancer progression, G3BP1 was overexpressed in MCF-7 human breast cancer cells, and the levels of EMT markers were then examined. The results showed that MCF-7 cells transiently transfected with G3BP1 exhibiting a mesenchymal-like phenotype (Figure 2A, left). As shown in Figure 2A on the right, expression of the epithelial markers E-cadherin and Keratin 19 was decreased, whereas that of the mesenchymal markers Fibronectin, Vimentin, Snail, Slug and ZEB1 was increased, indicating that overexpression of G3BP1 induced the EMT in MCF-7 cells. Moreover, overexpression of G3BP1 significantly promoted MCF-7 cell migration and invasion (Figure 2B).

In addition, we established two stably G3BP1-overexpressing clones (G3BP1(+)-1 and G3BP1(+)-2) and a Mock clone transfected with an empty vector. Consistently, the stably G3BP1-overexpressing clones exhibited a mesenchymal-like phenotype and enhanced migratory and invasion capacities compared with those of the Mock clone (Figure 2C and 2E). Moreover, expression of the epithelial markers E-cadherin and Keratin 19 was decreased and that of the mesenchymal markers Fibronectin, Vimentin, Snail, Slug and Zeb1 was increased, suggesting that overexpression of G3BP1 induced the EMT in MCF-7 cells (Figure 2D).

### Downregulation of G3BP1 suppresses the mesenchymal phenotype in MDA-MB-231 cells

**Figure 3:**
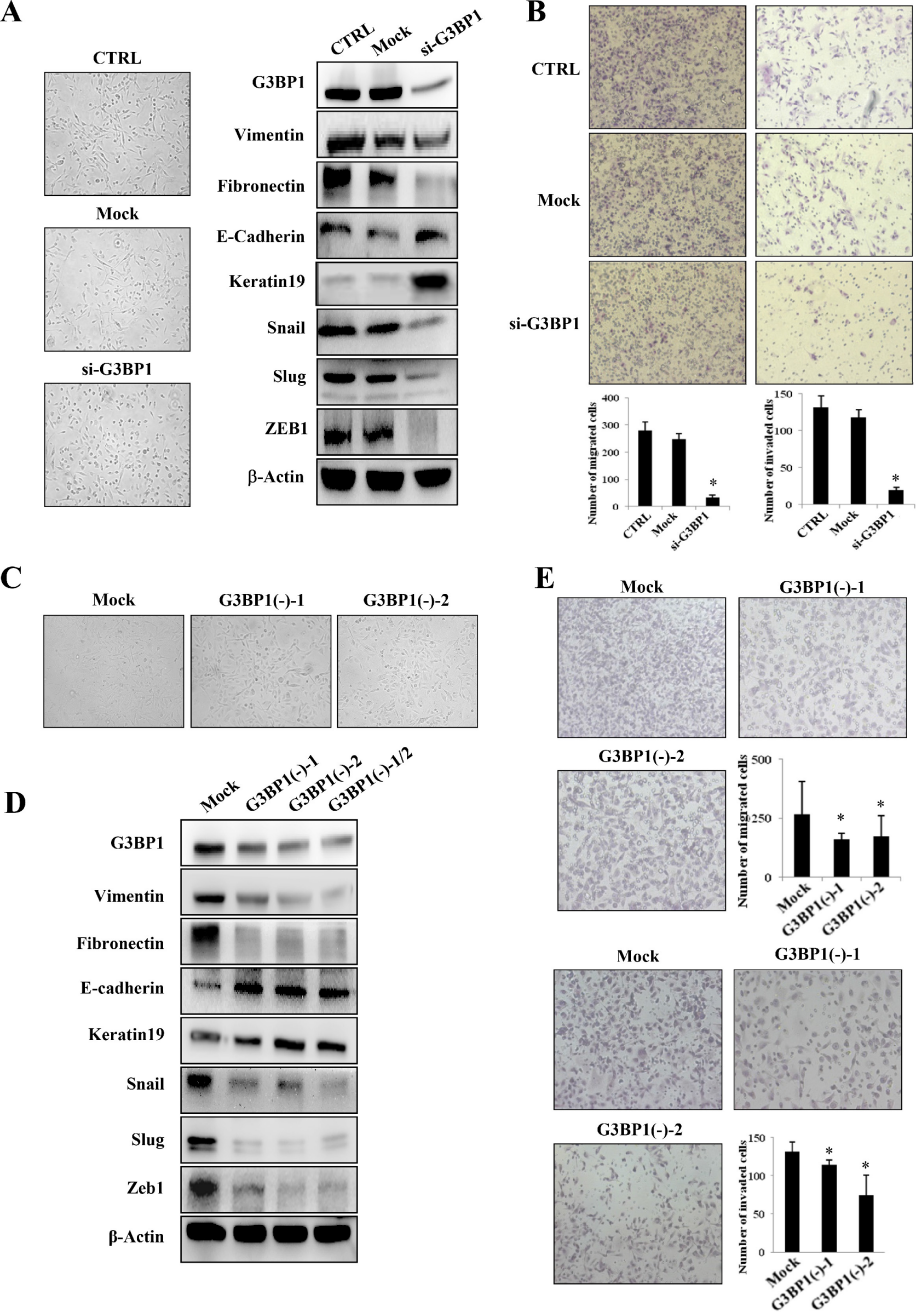
Silencing of G3BP1 suppresses the mesenchymal phenotype and inhibits the migration and invasion of MDA-MB-231 cells **A.** Changes in the morphology (left) and the expression of epithelial and mesenchymal markers (right) of MDA-MB-231 cells untreated (CTRL) or transiently transfected with NControl siRNA (Mock) or G3BP1 siRNA (si-G3BP1) (magnification, × 100). β-actin was used as a loading control. **B.** Transwell migration assay (left) and Matrigel invasion assay (right) using MDA-MB-231 cells (CTRL, Mock or G3BP1) (magnification, × 100). *P < 0.05 versus Mock. The data represent the means±s.d. of three independent experiments. **C.** Morphological changes in MDA-MB-231 cell lines stably expressing shG3BP1 (G3BP1(−)-1 or G3BP1(−)-2) or an empty vector (Mock) (magnification, × 100). **D.** Western blot of whole-cell extracts from MDA-MB-231 cells (Mock, G3BP1(−)-1, G3BP1(−)-2 or the mixture of these two clones (G3BP1(−)-1/2)) for epithelial and mesenchymal markers. β-actin was used as a loading control. **E.** Transwell migration assay (upper) and Matrigel invasion assay (lower) using MDA-MB-231 cells (Mock, G3BP1(−)-1 or G3BP1(−)-2) (magnification, × 100). *P < 0.05 versus Mock. The data represent the means±s.d. of three independent experiments.

The above results demonstrated that overexpression of G3BP1 induced the EMT in MDCK and MCF-7 cells. To verify whether these changes associated with the EMT were specifically induced by G3BP1, the G3BP1 gene was silenced using siRNA in highly metastatic MDA-MB-231 breast cancer cells. We found that G3BP1 knockdown decreased the levels of the mesenchymal markers and increased the levels of the epithelial markers (Figure 3A, right). Consistent with these changes of EMT markers, the G3BP1-silenced MDA-MB-231 cells exhibited tight cell clusters (Figure 3A, left) and decreased cell migration and invasion (Figure 3B). Next, we established two stably G3BP1 shRNA-expressing clones (G3BP1(−)-1 and G3BP1(−)-2) by transfecting the pGU6/neo-shG3BP1 or pGU6/neo-control vector into MDA-MB-231 cells. Consistently, the results indicated that the stably G3BP1-silenced clones exhibited an epithelial-like phenotype (Figure 3C); expression of the epithelial markers E-cadherin and Keratin 19 was increased and that of the mesenchymal markers Vimentin, Snail, Slug, Fibronectin and ZEB1 was decreased (Figure 3D). Moreover, the migratory and invasion capacities were significantly reduced (Figure 3E). Taken together, these results suggested that downregulation of G3BP1 suppressed the EMT.

### Smads activation and Slug upregulation mediated G3BP1-induced EMT

**Figure 4:**
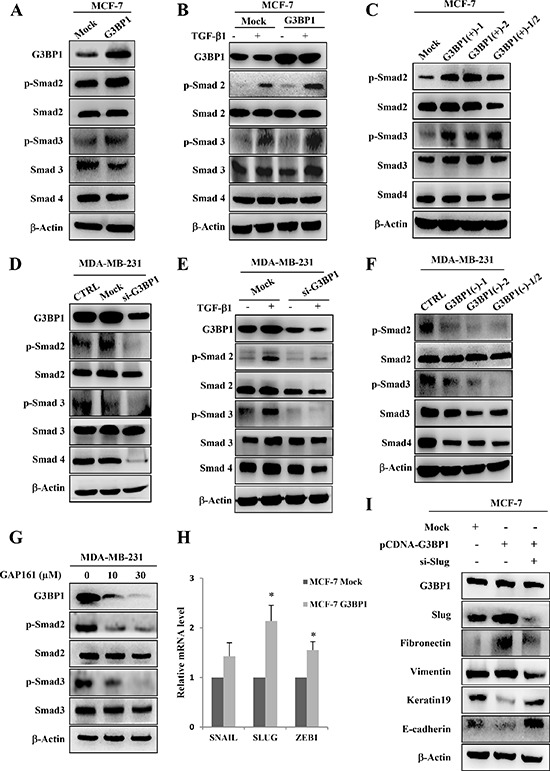
Smads activation and Slug upregulation mediate G3BP1-induced EMT **A.** Western blot of whole-cell extracts from MCF-7 cells transiently transfected with pCDNA3.1-control (Mock) or pCDNA3.1-G3BP1 (G3BP1) for G3BP1, phospho-Smad2 (p-Smad2), total Smad2 (Smad2), phospho-Smad3 (p-Smad3), total Smad3 (Smad3), and Smad4. **B.** G3BP1 enhanced the TGF-β1-induced phosphorylation of Smad2 and Smad3. MCF-7 cells were transfected with either pCDNA3.1-control (Mock) or pCDNA3.1-G3BP1 (G3BP1) with or without TGF-β1 (5 ng/mL) for 24 h. **C.** The levels of p-Smad2 and p-Smad3 in MCF-7 cells stably expressing an empty vector (Mock), G3BP1 (G3BP1(+)-1 or G3BP1(+)-2) or the mixture of these two clones (G3BP1(+)-1/2). **D.** The siRNA-mediated silencing of G3BP1 attenuated the phosphorylation of Smad2 and Smad3 in MDA-MB-231 cells. **E.** The siRNA-mediated silencing of G3BP1 attenuated the TGF-β1-induced phosphorylation of Smad2 and Smad3. MDA-MB-231 cells were transfected with either NControl siRNA (Mock) or G3BP1 siRNA (si-G3BP1) with or without TGF-β1 (5 ng/mL) for 24 h. **F.** The phosphorylation status of Smad2 and Smad3 in MDA-MB-231 cells stably expressing an empty vector (Mock), shG3BP1 (G3BP1(−)-1 or G3BP1(−)-2) or the mixture of these two clones (G3BP1(−)-1/2). β-actin was used as a loading control. **G.** The phosphorylation of Smad2 and Smad3 was attenuated in the MDA-MB-231 cells after treatment with GAP161 for 48 h. **H.** The mRNA expression levels of SNAIL, SLUG, ZEB1 and GAPDH in MCF-7 cells transfected with pCDNA3.1-control (Mock) or pCDNA3.1-G3BP1 (G3BP1) were measured via RT-PCR. *P < 0.05 versus Mock. The data represent the means±s.d. of three independent experiments. GAPDH was used as an internal control. **I.** Knocking down Slug abolished the G3BP1-induced changes in the expression of epithelial and mesenchymal markers. β-actin was used as a loading control.

To further explore the mechanism of the G3BP1-induced EMT, we focused on the Smad signaling pathway, which is known to play a major role in EMT-induced cancer progression and metastasis. We hypothesized that G3BP1 participates in the Smad signaling pathway to mediate the EMT. To address this hypothesis, we examined the effect of G3BP1 on the Smad signaling pathway by either overexpressing G3BP1 in MCF-7 cells or silencing G3BP1 in MDA-MB-231 cells. Transiently increasing G3BP1 expression caused a dramatic activation of Smad2/3 in MCF-7 cells compared with Mock-treated MCF-7 cells (Figure 4A). To further confirm that G3BP1 activates Smads, we used siRNA or shRNA to knock down G3BP1 expression. Transfection with G3BP1 siRNA resulted in a substantial decrease in Smad2/3 phosphorylation in MDA-MB-231 cells (Figure 4D), suggesting that G3BP1 was required for Smad activation. Similarly, the phosphorylation of Smad2 and Smad3 was increased in stably G3BP1-overexpressing MCF-7 cell clones (Figure 4C), whereas it was decreased in stably G3BP1-downregulated MDA-MB-231 cell clones (Figure 4F).

Next, TGF-β was also introduced into different human breast cancer cell lines. We found that Smad2/3 phosphorylation was significantly increased in both TGF-β-treated MCF-7/G3BP1(+) and MCF-7/Mock cells compared with TGF-β-untreated cells (Figure 4B). This stimulatory effect was dramatically enhanced in G3BP1-overexpressing MCF-7/G3BP1(+) cells compared with MCF-7/Mock cells. However, the TGF-β-mediated increase in Smad2/3 phosphorylation was detected in MDA-MB-231/Mock cells but not in MDA-MB-231/G3BP1(−) cells (Figure 4E). Moreover, GAP161, a G3BP1-targeting peptide that inhibits G3BP1 and displays promising anti-cancer activity [16], was applied to MDA-MB-231 cells. Following the downregulation of G3BP1 in response to GAP161, the phosphorylation of Smad2 and Smad3 was decreased (Figure 4G). These data indicated that G3BP1 participated in the regulation of Smad signaling pathway. To extend our finding that G3BP1 mediated the activation of the Smad signaling pathway, we investigated whether G3BP1-mediated EMT depends on the absence or presence of TGF-β. As expected, we found that G3BP1 knockdown abolished the TGF-β-induced EMT in MCF-7 cells (Figure S3A). In addition, knockdown of G3BP1 significantly inhibited TGF-β-induced cell migration and invasion (Figure S3B) which further suggested an important role of G3BP1 in the regulation of TGF-β-induced EMT. These results also indicated that the regulation of G3BP1 on EMT in MCF-7 cells didn't depend on the absence or presence of TGF-β.

Given that activated Smads could upregulate EMT associated transcription factors Snail, Slug and Zeb1 expression [19], we wondered which transcription factors contributed to G3BP1-induced EMT. We then examined the translocation of Smads induced by G3BP1 via western blotting in MCF-7 cells. As shown in Figure S4, G3BP1 promoted the shuttle of Smads from cytoplasm to nucleus which agreed with the changes in the expression of Snail1, Slug and ZEB1 (Figure 2A, right). Moreover, upregulation of G3BP1 promoted the transcriptional expression of SNAIL, SLUG and ZEB1 (Figure 4H). The most remarkable change in mRNA expression was SLUG, thus we assumed Slug plays the most important role in G3BP1-induced EMT. Furthermore, we co-transfected siRNA-Slug and pCDNA3.1-G3BP1 in MCF-7 cells, and found that knockdown of Slug could reverse G3BP1 mediated EMT (Figure 4I).

Taken together, these results demonstrate that G3BP1 overexpression contributes to the activation of Smads, which induces EMT transcription factors expression. Slug plays the most important role in G3BP-induced EMTs in MCF-7 cells.

### G3BP1 interacts with smads

**Figure 5:**
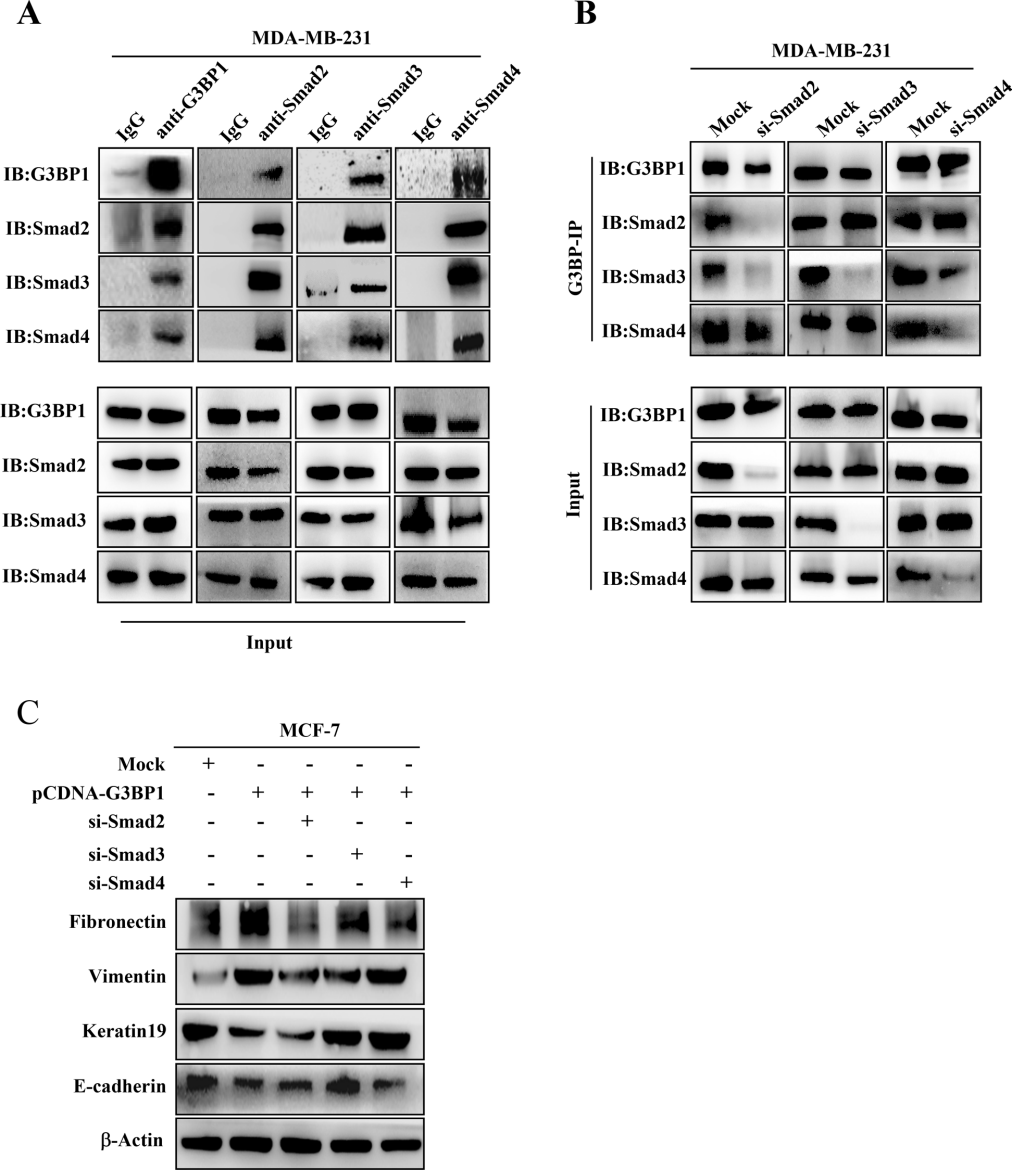
G3BP1 interacts with Smads **A.** The interaction between G3BP1 and Smad2, Smad3 and Smad4 based on endogenous co-immunoprecipitation in MDA-MB-231 cells. Whole-cell extracts were immunoprecipitated using antibodies against G3BP1, Smad2, Smad3 or Smad4 and then immunoblotted for the indicated proteins. **B.** Whole-cell extracts from MDA-MB-231 cells were immunoprecipitated and immunoblotted using the indicated antibodies. The cells were transfected with siRNA targeting Smad2, Smad3 or Smad4. **C.** Changes in the expression levels of epithelial and mesenchymal markers were measured via western blot of MCF-7 cells transiently transfected with pCDNA3.1-control (Mock) or pCDNA3.1-G3BP1 together with siRNA targeting Smad2, Smad3 or Smad4. β-actin was used as a loading control.

Because G3BP1 affected the phosphorylation of Smad2 and Smad3, we hypothesized that G3BP1 plays a direct role in recruiting Smad signaling molecules. Endogenous interactions between G3BP1 and Smad2/3/4 were confirmed by co-immunoprecipitation assay in MDA-MB-231 cells (Figure 5A). These interactions were also found in MCF-7 cells (data not shown). The results of the reciprocal co-immunoprecipitation suggested that G3BP1 activated Smad signaling by recruiting the Smad2/3 complex. To identify which component directly bound to G3BP1, we repeated co-immunoprecipitation assay using various antibodies and lysates from MDA-MB-231 cells transfected with siRNA targeting Smad2, Smad3 or Smad4. The results indicated that knockdown of Smad2 and Smad4, but not Smad3, disrupted the G3BP1-Smad interactions, suggesting that G3BP1 directly binds to the Smad2/4 complex (Figure 5B). Together, our data suggest that G3BP1 acts as a novel binding partner to the Smad complex that activates Smad signaling by recruiting the Smad2/4 complex.

We next determined whether Smads were necessary for G3BP1-induced EMT. MCF-7 cells were transfected with pCDNA3.1-G3BP1 together with siRNA targeting Smad2, Smad3 or Smad4. The results revealed that Smad3 knockdown almost completely reversed the G3BP1-mediated EMT by increasing the expression of E-cadherin and Keratin19 and decreasing the expression of Vimentin and Fibronectin (Figure 5C). Transfection with Smad2 or Smad4 siRNA partially inhibited the G3BP1-mediated EMT (Figure 5C). These data indicated that Smads, particularly Smad3, were essential for the G3BP1-mediated EMT.

### Downregulation of G3BP1 inhibits tumor metastasis *in vivo*

**Figure 6:**
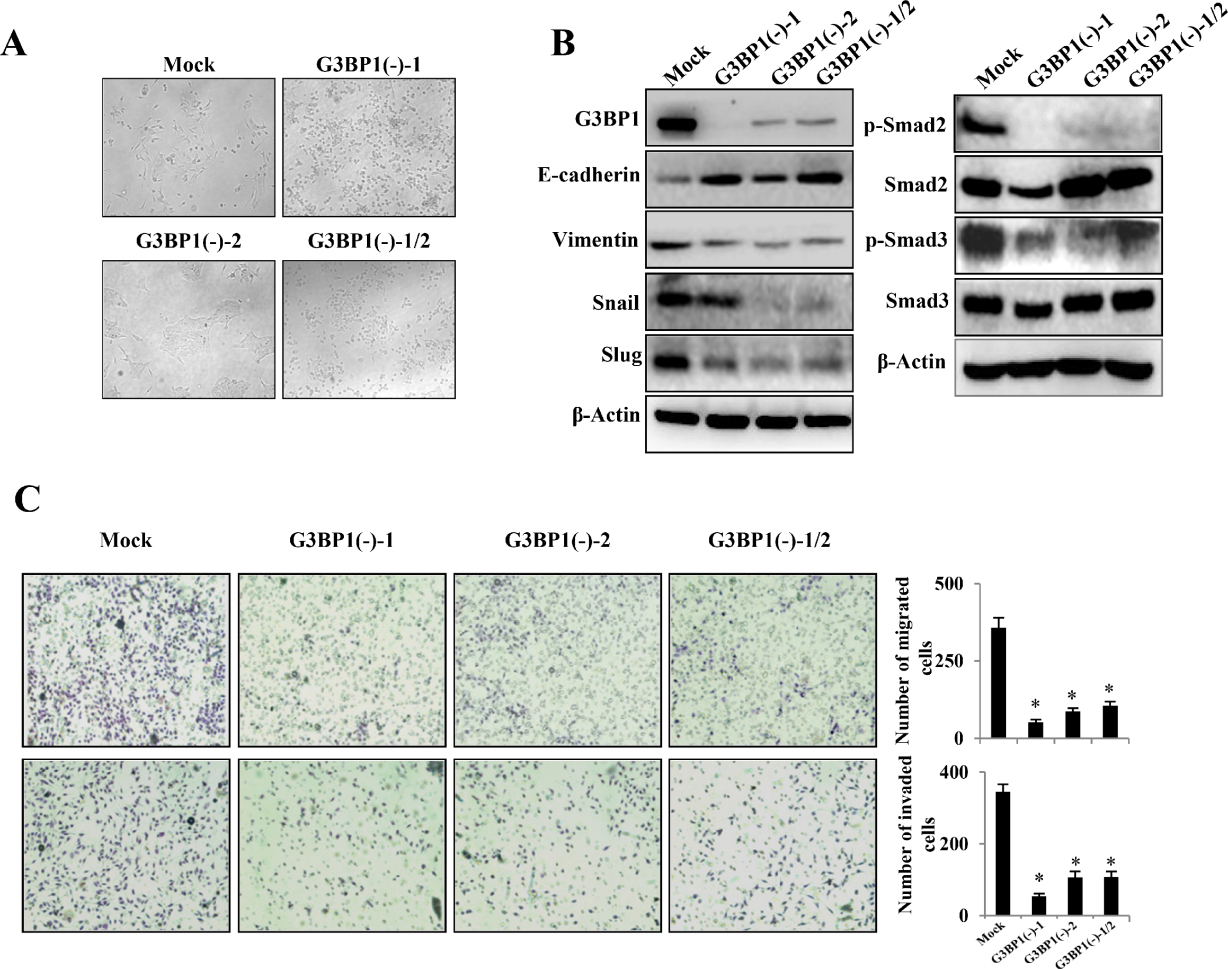
Downregulation of G3BP1 suppresses the mesenchymal phenotype of 4T1 cells **A.** Morphological changes in 4T1-luc cell lines stably expressing mus-shG3BP1 (G3BP1(−)-1 or G3BP1(−)-2) or an empty vector (Mock) (magnification, × 100). **B.** Western blot of whole-cell extracts from 4T1 cells (Mock, G3BP1(−)-1, G3BP1(−)-2 or the mixture of these two clones (G3BP1(−)-1/2)) for epithelial and mesenchymal markers (left), phospho-Smad2 (p-Smad2), total Smad2 (Smad2), phospho-Smad3 (p-Smad3) and total Smad3 (Smad3) (right). β-actin was used as a loading control. **C.** Transwell migration assay (upper) and Matrigel invasion assay (lower) using 4T1 cells (Mock, G3BP1(−)-1, G3BP1(−)-2 or G3BP1(−)-1/2) (magnification, × 100). *P < 0.05 versus Mock. The data represent the means±s.d. of three independent experiments.

To determine whether G3BP1 affected tumor growth and lung metastasis in 4T1 cells *in vivo*, we constructed two stably G3BP1 shRNA-expressing clones (G3BP1(−)-1 and G3BP1(−)-2) by transfecting the pGU6/hygro-mus-shG3BP1 or pGU6/hygro-control vector into murine mammary carcinoma 4T1-luc cells. Silencing G3BP1 reduced the mesenchymal phenotype (Figure 6A and 6B, left), the migratory capacity and the invasiveness of 4T1 cells (Figure 6C). As shown in Figure 6B on the right side, downregulation of G3BP1 also attenuated the activity of the Smad signaling pathway in 4T1 cells.

**Figure 7:**
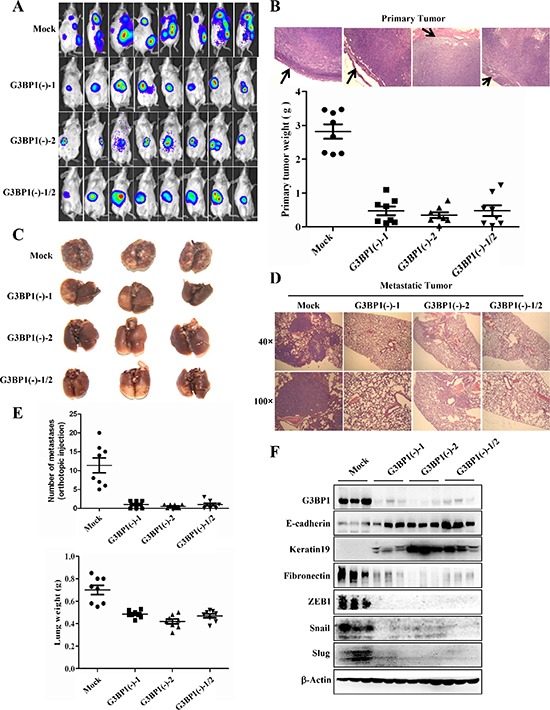
Downregulation of G3BP1 inhibits tumor metastasis *in vivo* **A.** BLI of the indicated cell lines. A total of 5.0 × 10^5^ 4T1-luc cells in 0.1 mL of PBS were orthotopically injected into the mammary fat pad of 6- to 8-week-old female BALB/c mice (*n* = 8 each). **B.** Representative images of primary tumor margins stained with hematoxylin and eosin (upper panel) (magnification, × 100). The arrows indicate the invasive front. The weight of the primary tumor from the indicated cell lines is shown in the lower panel. **C.** Representative lungs were harvested via necropsy after orthotopic injection (*n* = 3). **D.** Representative images of hematoxylin and eosin staining of lung sections (magnification, × 40 upper, × 100 lower). **E.** The number of metastatic nodules (upper panel) and the lung weight (lower panel) were used to evaluate metastasis to the lungs. The number of metastatic nodules and the lung weight are presented as the means±s.d. **F.** Tumors from mice sacrificed 41 days after injection of 4T1-luc cells (Mock, G3BP1(−)-1, G3BP1(−)-2 or G3BP1(−)-1/2 (*n* = 3 each)) were analyzed for the expression levels of EMT markers.

4T1 cells are syngeneic to BALB/c mice. Both 4T1-luc/G3BP1(−)-1 and 4T1-luc/G3BP1(−)-2 cells and a mixture of 4T1-luc/G3BP1(−)-1/2 cells were implanted into the mammary fat pad of mice (BALB/c) to examine their ability to form metastatic tumors. A bioluminescence imaging (BLI) system was applied to examine the primary tumor burden and the development of lung metastasis *in vivo* after the injection of 4T1 cells [20]. At 41 days after 4T1 cell inoculation, the 4T1-luc/Mock tumors induced significantly increased bioluminescent signals at primary and secondary sites (primarily in the lungs), whereas the G3BP1-knockdown 4T1/G3BP1(−) tumors were smaller at the primary site and were noninvasive at secondary sites (Figure 7A). As shown in Figure 7B, the average weight of the 4T1/Mock tumors was much larger than that of the 4T1-luc/G3BP1(−)-1, 4T1-luc/G3BP1 (−)-2 and 4T1-luc/G3BP1(−)-1/2 tumors, suggesting that G3BP1 significantly promoted tumorigenesis and growth in this orthotopic breast cancer model and that G3BP1 downregulation suppressed primary tumor growth. Furthermore, the 4T1-luc/G3BP1(−) tumors formed no visible metastases, whereas the hosts of the 4T1-luc/Mock cells exhibited an average of 12 metastatic nodules in the lungs (Figure 7C and 7E, upper).

Metastases in the lungs of mice carrying tumors were confirmed via H&E staining and the lung weight (Figure 7D and 7E, lower). We found many micrometastases in the lungs of mice carrying Mock tumors, in stark contrast with the few micrometastases detected in the lungs of mice carrying G3BP1(−)-1, G3BP1(−)-2 or G3BP1(−)-1/2 tumors. Next, we confirmed that the G3BP1(−)-1, G3BP1(−)-2, G3BP1(−)-1/2 and Mock tumors continued to maintain their epithelial or mesenchymal phenotypes *in vivo*. As shown in Figure 7F, the Mock tumors maintained high expression of G3BP1 post-implantation, whereas the G3BP1(−)-1, G3BP1(−)-2 and G3BP1(−)-1/2 tumors displayed negligible expression of G3BP1. The G3BP1-downregulated tumors also displayed positive expression of E-cadherin and Keratin19, whereas the Mock tumors were positive for Fibronectin. Furthermore, expression of the transcription factors that regulate E-cadherin, such as Snail, Slug and ZEB1, was significantly decreased in G3BP1-downregulated tumors. Taken together, these data suggested that the downregulation of G3BP1 suppressed 4T1 tumor growth and metastasis *in vivo*.

## DISCUSSION

We previously reported that G3BPs played an important role in inducing the invasion and metastasis of human lung cancer H1299 cells via the Src/FAK-associated signaling pathway [17]. In this study, the overexpression of G3BP1 induced the EMT in a model of MDCK cells. Although G3BP1 was overexpressed in many tumor samples compared to healthy tissue [21], the up-regulation of G3BP1 promoted an invasive and metastatic phenotype in non-metastatic breast cancer MCF-7 cells, whereas the down-regulation of G3BP1 suppressed this invasive and metastatic phenotype in metastatic MDA-MB-231 breast cancer cells. Despite previous reports demonstrating that G3BP1 was associated with cell motility and invasion [14, 22], our study provided the first evidence that G3BP1 mediates the EMT in breast cancer cells.

The EMT was initially described in the early 1980s by Elizabeth Hay [23]. Although a variety of factors and signals have been shown to induce the EMT during embryogenesis and tumorigenesis, TGF-β was the first inducer demonstrated to activate the EMT in mammary epithelial cells [24]. In response to TGF-β, TβR-I is phosphorylated by TβRII, leading to the C-terminal phosphorylation of Smad2 and Smad3, which then form a trimer with Smad4 and translocate into the nucleus to associate and cooperate with DNA-binding transcription factors to regulate target gene transcription [25]. In this study, we found that G3BP1 involved in the activation of Smad signaling pathway. Overexpression of G3BP1 enhanced the phosphorylation of Smad2 and Smad3, whereas downregulation of G3BP1 attenuated the phosphorylation of Smads in the presence or absence of exogenous TGF-β1. TGF-β1 plays an important role in inducing EMT, we also found that silencing G3BP1 abrogated the TGF-β-induced EMT process (Figure S3A, B), which indicated that G3BP1 is crucial for TGF-β-induced EMT.

The RT-PCR results revealed that the mRNA expression levels of TGFBR2 and TGFB1 were upregulated in G3BP1-overexpressing MCF-7 cells (Figure S2). Silencing G3BP1 attenuated the activation of Smad2/3 without altering the expression of TβRII or TGF-β1. We speculated that this enhanced expression of TβRII and TGF-β1 occurred due to feedback activation in response to the G3BP1-mediated activation of Smad2/3.

The activated Smads induce EMT via three families of transcription factors, resulting in repression of epithelial marker gene expression and activation of mesenchymal gene expression. We found that overexpressed G3BP1 increased nuclear localized activated Smads, which may promoted the transcriptional expression of SNAIL, SLUG and ZEB1. And Slug played the most important role as knockdown of Slug inhibited the G3BP1-induced changes in the expression of marker genes (Figure 4I). E-cadherin repressor Slug is reported to overexpress in numerous cancer and promote cancer cells to become motile and invasive through downregulating epithelial markers and upregulating mesenchymal markers [26]. As Smad complex binds to the promoter of Slug and induces expression of Slug [27], we speculated that G3BP1 promoted the EMT mainly via the activated Smads through the upregulation of Slug.

Although Smad2 and Smad3 are key elements in the Smad signaling pathway, they may play distinct roles in the induction of the EMT in breast cancer. A recent study reported that the activation of Smad3, but not Smad2, was essential to induce the EMT in normal mammary epithelial cells [28]. Likewise, Smad2 knockdown strikingly enhances metastatic potential, whereas the loss of Smad3 prolongs the latency and delays the growth of bone metastases in MDA-MB-231 cells [29]. Those reports indicate that Smad3 is more critical than Smad2 for induction of the EMT during breast cancer. In addition, Smad4 is necessary for the TGF-β-induced EMT and metastasis of breast cancer cells to bone [30]. We got similar result shown in Figure S1 that Smad3 knockdown strikingly affected the expression of EMT marker genes. To validate which Smad is critically responsible for the G3BP1-induced EMT, we used multiple Smad-targeted siRNAs to transiently knock down various Smads. We also found that knockdown of specific Smads influenced the expression of various EMT markers during G3BP1 overexpression. G3BP1 failed to alter the expression of these EMT markers following Smad knockdown, verifying that G3BP1 acts upstream of the Smads and induces the EMT via the Smad signaling pathway. As shown in Figure 5C, Smad3 knockdown nearly abolished the G3BP1-mediated changes in the expression of epithelial and mesenchymal markers, and silencing Smad2/4 partially blocked these changes. We speculate that Smad3 is more crucial than Smad2/4 for the G3BP1-induced EMT in breast cancer.

The *in vivo* co-immunoprecipitation assay indicated that G3BP1 co-precipitated with Smad2, Smad3 and Smad4 and that these interactions similarly occurred via the Smad complex. As noted above, G3BP1 may interact with the Smad2/4 and Smad3/4 complexes. Individually silencing the Smads to elucidate their direct interactions with G3BP1 suggested that G3BP1 binds to Smad2/4. G3BP1, which is necessary for the phosphorylation of Smad2/3, might act as a novel co-factor to regulate the phosphorylation status of Smads. It will be interesting to investigate how the co-factor G3BP1 acts on Smads.

Aside from these *in vitro* experiments on the G3BP1-induced EMT, we evaluated the G3BP1-mediated regulation of primary tumor metastasis *in vivo*. Our data indicated that G3BP1 downregulation suppressed primary tumor growth and lung metastasis of mouse 4T1 breast cancer cells. Thus, G3BP1 knockdown inhibited breast cancer metastasis to the lungs. We have previously demonstrated that the RasGAP-derived peptides 38GAP and GAP159 potentiate the cytotoxicity of cisplatin to HCT116 cells [16, 31, 32]. Herein, GAP161 inhibited the expression of G3BP1 and attenuated the phosphorylation of Smad2 and Smad3 in a dose-dependent manner, consistent with the results of G3BP1 siRNA treatment (Figure 4D). Thus, GAP161 may represent a novel therapeutic agent for breast cancer metastasis.

The EMT is a complex process that is associated with the initiation of cancer stem cells and increases in cell aggressiveness, invasion and metastasis, drug resistance and disease recurrence in breast cancer and other carcinomas regulated by the Smad signaling pathway [33–36]. In the present study, we provided the first evidence that G3BP1 induces the EMT via the Smad signaling pathway. Moreover, our study not only elucidated the mechanism by which G3BP1 induces the EMT but also provided a potential therapeutic target for the invasion and metastasis of breast cancer.

## METHODS

### Cell culture

The Madin-Darby canine kidney (MDCK), MDA-MB-231 (human breast cancer), MCF-7 (human breast cancer), human embryonic kidney (HEK)293T and 4T1-luc (mouse breast cancer) cell lines were purchased from the Cell Culture Center of Peking Union Medical College (Beijing, China). The MDCK, MCF-7 and HEK-293T cells were cultured in DMEM (HyClone, Logan, UT, USA) supplemented with 10% fetal bovine serum (TBD, Tianjin, China). The 4T1-luc cells were cultured in 1640 medium (HyClone) supplemented with 10% fetal bovine serum (TBD). The MDA-MB-231 cells were cultured in L-15 medium (HyClone) supplemented with 10% fetal bovine serum (Gibco, US). All cultures were routinely tested for mycoplasma and for the retention of their respective morphology and growth characteristics.

### Plasmids and cell lines

pCDNA3.1-G3BP1 was generated via PCR and confirmed via sequencing analysis. Stable cell lines were selected using hygromycin. G3BP1 shRNAs were synthesized and cloned into the pGPU6/Hygro vector by Shanghai GenePharm (Shanghai, China) as described previously [17]. Stable transfectants were identified via selection using G418.

### siRNA interference and transfection

The siRNA sequences used were synthesized by Ribobio Technology (Guangzhou, China). The siRNA sequences were as follows: G3BP1, 5′-GCAA CAGUAUUUCGGUAUAdTdT-3′; Smad2, 5′-GGUGA AGAAGCUAAAGAAAdTdT-3′; Smad3, 5′-CCGCAU GAGCUUCGUCAAAdTdT-3′; Smad4, 5′-GCC AUAGU GAAGGACUGUUdTdT-3′; SLUG, 5′-GCAUUUGCA GACAGGUCAAdTdT-3′ and control Mock siRNA, 5′-UUCUCCGAACGUGUCACGUdTdT-3′. Cells at 40–60% confluence in six-well culture dishes were transfected with siRNA using Lipofectamine RNAiMAX (Invitrogen, Carlsbad, CA, USA) according to the manufacturer's instructions. Alternatively, cells at 40–60% confluence were transiently transfected with the indicated plasmid using Lipofectamine 2000 (Invitrogen, Carlsbad, CA, USA) according to the manufacturer's instructions.

### Reagents and antibodies

TGF-β1 was purchased from R&D Systems (Minneapolis, MN, USA). The cell-permeable peptide GAP161 was synthesized by Wuhan KatyGen Pharmaceuticals (Hubei, China). These peptides were dissolved in deionized water at a final concentration of 10 mM and stored at −80°C until further use. The antibodies and their suppliers included the following: G3BP1 (TT-Y) for immunoprecipitation and G3BP1 (H-10) for immunoblot from Santa Cruz Biotechnology (Santa Cruz, CA, USA); E-cadherin from BD Transduction Laboratories (Lexington, KY, USA); Keratin 17/19, Vimentin, Slug, Snail, ZEB1, Smad2, Smad3, phospho-Smad2 (Ser465/467), phospho-Smad3 (Ser423/425) and β-actin from Cell Signaling Technology (Danvers, MA, USA); and Fibronectin from Abcam (Cambridge, MA, USA).

### Immunoprecipitation and western blot

Cell supernatants were harvested and lysed in RIPA buffer (Cell Signaling Technology) 48 h after treatment, and the protein concentration was determined using an Enhanced BCA Protein assay kit (Beyotime, Jiangsu, China). Then, 1 mg of total protein was subjected to immunoprecipitation using the appropriate antibodies as indicated overnight at 4°C with gentle agitation, followed by incubation in Protein A/G PLUS-Agarose (Santa Cruz, CA, USA) for 4–6 hours at 4°C. For western blot, the precipitated proteins or whole-cell lysates were subjected to SDS-PAGE and transferred to polyvinylidene difluoride membranes (Millipore Corp., Bedford, MA, USA). The membranes were then probed with the indicated antibodies, and the immunoreactive bands were developed using an ECL detection system (Millipore, Billerica, MA, USA).

### Immunofluorescence

MDCK cells and their derivatives were fixed in 4% paraformaldehyde, permeabilized in 0.1% Triton-X100 and blocked in 5% BSA. The slides were then incubated in primary antibodies overnight at 4°C followed by Alexa Fluor 488- and/or Alexa Fluor 594-conjugated secondary antibodies (Invitrogen). The nuclei were stained with Hoechst 33258. Images were obtained under a fluorescence microscope (TE2000-U, Nikon) and were analyzed using the corresponding software.

### Cell migration and invasion assays

Cell migration was measured according to the ability of the cells to migrate across a transwell filter (8-μm pores, Costar, Cambridge, MA, USA), whereas the invasion capability was measured according to the ability of the cells to migrate across a transwell filter coated with 2.5 mg/mL Matrigel (BD Biosciences, Bedford, MA, USA). Cells suspended in serum-free DMEM or L-15 medium were added to the upper chamber, and DMEM or L-15 medium containing 20% fetal bovine serum was added to the lower chamber. After the cells were incubated at 37°C for 24 h, the cells that migrated to the lower side of the upper chamber were stained with hematoxylin, and the cells per microscopic field (× 100) were counted under a microscope.

### *In vivo* tumor experiment

The 6- to 8-week-old female mice (BALB/c) were purchased from the Institute for Experimental Animals of the Chinese Academy of Medical Sciences (Beijing, China). The 4T1-luc cells were suspended at a final concentration of 5.0 × 10^6^ cells/mL in PBS, and 5.0 × 10^5^ 4T1-luc cells in 0.1 mL of PBS were injected into the second mammary fat pad. On day 41, the mammary tumors and lungs were removed and weighed. The lungs were fixed in formalin overnight before evaluating lung metastasis, and the numbers of metastatic nodules on the lung surface were counted. For histological examination, the primary tumor was sectioned into two parts. One half was used for western blot analysis; the other half and the lungs were fixed in formalin and embedded in paraffin blocks for slicing into thin sections. The paraffinized sections were stained with hematoxylin and eosin (H&E) according to standard protocols. The stained sections were photographed using a Leica microscope (Leica, Wetzlar, Germany).

### Optical imaging and histological analysis

On day 41, before euthanizing the mice, an optical molecular imaging system was used to evaluate primary tumor growth and metastasis. The luciferase substrate D-luciferin (150 mg/kg) was injected intraperitoneally, and the animals were placed into a warmed stage inside the camera box of an IVIS Imaging System (Xenogen, Alameda, CA, USA) to observe the tumor and the lungs. Measurements were performed as previously described [37].

## SUPPLEMENTARY MATERIALS AND METHODS FIGURES


