# Supplementary material for: Involvement of Ras GTPase-activating protein SH3 domain-binding protein 1 in the epithelial-to-mesenchymal transition-induced metastasis of breast cancer cells via the Smad signaling pathway
Source: Oncotarget. 2015 Apr 20;6(19):17039–53. doi: 10.18632/oncotarget.3636 (PMC4627290; doi:10.18632/oncotarget.3636)
Supplement: Supplementary file 1 [file oncotarget-06-17039-s001.pdf]

SUPPLEMENTARY MATERIALS AND METHODS

RT-PCR and real-time PCR

Total RNA was isolated from MCF-7 and MDA-MB-231 cells using TRIzol reagent (Invitrogen, Carlsbad, CA, USA), and cDNA was generated using a Transcriptor First Strand cDNA Synthesis Kit (Roche, Mannheim, Germany). RT-PCR was performed using PrimeSTAR® HS DNA Polymerase (TaKaRa, Dalian, China). The quantitative RT-PCR reactions were performed using FastStart TaqMan Probe Master (ROX) (Roche, Mannheim, Germany). All samples were processed and measured using the ABI7500Fast Real-Time PCR Detection System (Applied Biosystems, Foster City, CA, USA). TaqMan probes for hG3BP1, hTGFBR1, hTGFBR2, hTGFB1, hSMAD4, hSNAIL, hSLUG, hZEB1 and hGAPDH were purchased from ABI. GAPDH was used as an internal control.

Nuclear and cytoplasmic extract preparations

The cultured cells were lysed using a Nucl-Cyto-Mem preparation Kit (Applygen Technologies Inc., Beijing, China) following the manufacturer's instructions. Cells was harvested and added to CER for 15 minutes in the ice bath. Then centrifuged at 8,000 g for 5 minutes at 4°C. Add MER to the supernatants for 5 minutes in the ice bath and centrifuged at 14,000 rpm for 30 minutes at 4°C. The supernatants were collected as the cytoplasmic fraction. The pellet was resuspended in NER and votexed vigorously. Repeat the process. The pellet was collected as the nuclear fraction. The separated cytoplasmic and nuclear extracts were prepared for immunoblotting.

SUPPLEMENTARY FIGURES

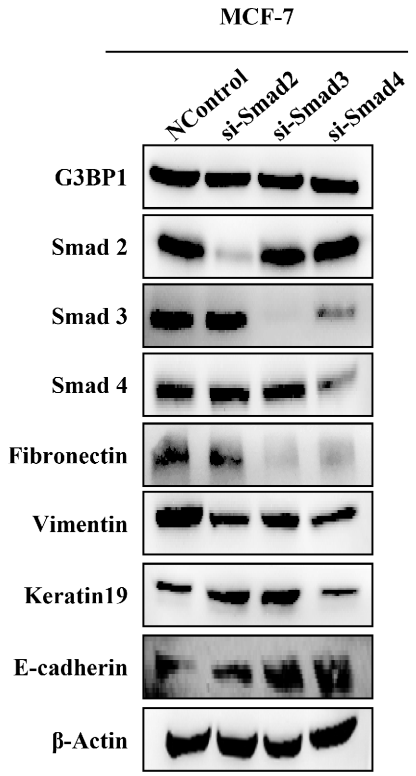

Supplementary Figure 1: Downregulation of Smads influences the EMT markers expression in MCF-7 cells. Changes in the expression levels of epithelial and mesenchymal markers were measured via western blot of MCF-7 cells transiently transfected with NControl-siRNA or siRNA targeting Smad2, Smad3 or Smad4. β-Actin was used as a loading control.

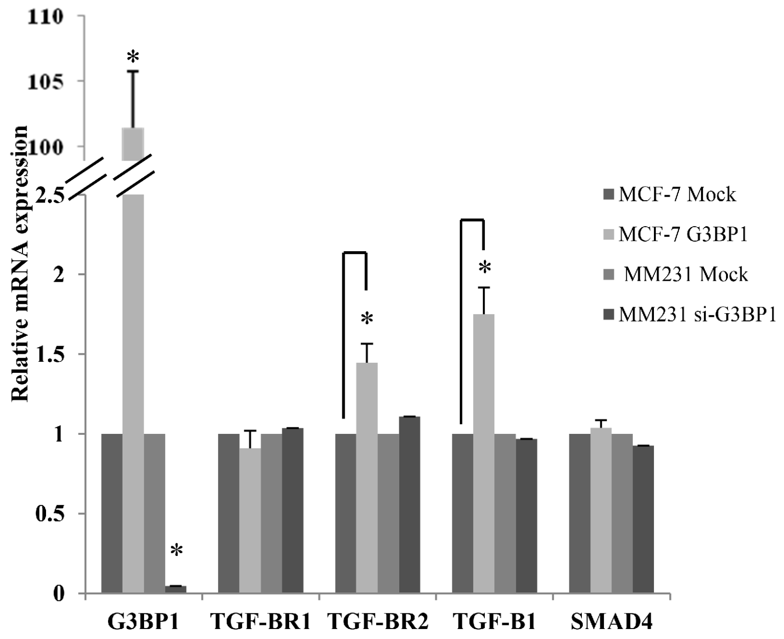

Supplementary Figure 2: Overexpression of G3BP1 increases the mRNA expression of TGFBR2 and TGFB1 in MCF-7 cells. The mRNA expression levels of hG3BP1, hTGFBR1, hTGFBR2, hTGFB1, hSMAD4 and hGAPDH in MCF-7 cells transfected with pCDNA3.1-control (Mock) or pCDNA3.1-G3BP1 (G3BP1) and in MDA-MB-231 cells transfected with NControl-siRNA (Mock) or G3BP1 siRNA (si-G3BP1) were measured via RT-PCR. *$P < 0.05$ versus Mock. The data represent the means±s.d. of three independent experiments. GAPDH was used as an internal control.

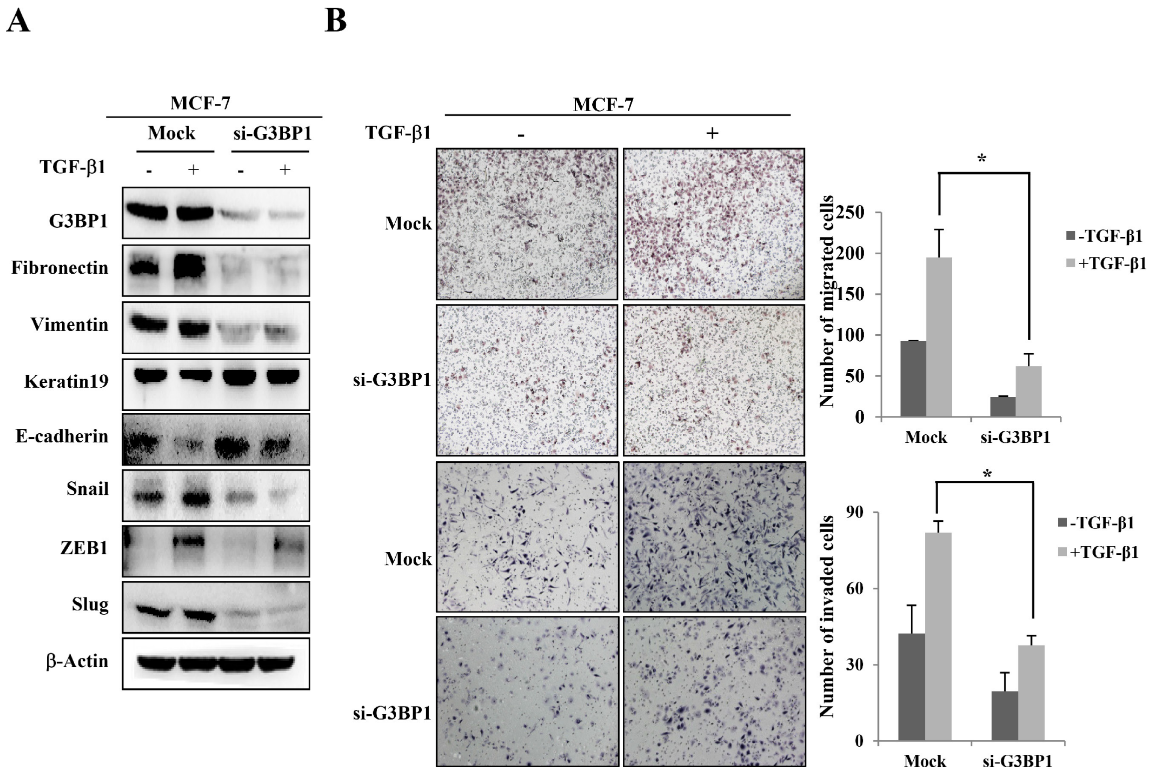

Supplementary Figure 3: Knockdown of G3BP1 inhibites TGF-β-induced EMT. A. Knocking down G3BP1 abolished the TGF-β1-induced changes in the expression of epithelial and mesenchymal markers. β-actin was used as a loading control. **B.** Transwell migration assay (upper) and Matrigel invasion assay (lower) using MCF-7 cells (Mock, G3BP1) with or without TGF-β1 (5 ng/mL) for 24 h (magnification, × 100). *$P < 0.05$ versus Mock. The data represent the means±s.d. of three independent experiments.

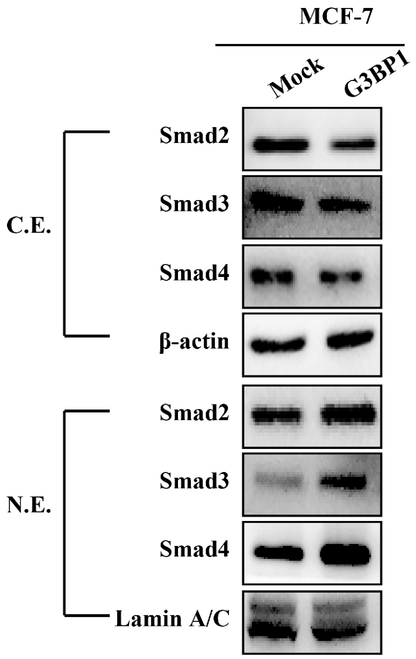

Supplementary Figure 4: Overexpression of G3BP1 promotes the shuttle of Smads from cytoplasm to nucleus. Western blot of nuclear and cytosolic extracts from MCF-7 cells transiently transfected with pCDNA3.1-control (Mock) or pCDNA3.1-G3BP1. β-actin and Lamin A/C were used as loading controls, respectively.
